# Anaesthetic Management of Two Patients with Pompe Disease for Caesarean Section

**DOI:** 10.1155/2014/650310

**Published:** 2014-03-20

**Authors:** I. J. J. Dons-Sinke, M. Dirckx, G. P. Scoones

**Affiliations:** Department of Anesthesiology, Erasmus MC-Sophia, Postbus 2060, 3000 CB Rotterdam, The Netherlands

## Abstract

The introduction of enzyme replacement therapy and the resultant stabilisation or improvement in mobility and respiratory muscle function afforded to patients with late-onset Pompe may lead to an increased number of Pompe patients prepared to accept the challenges of parenthood. In this case report, we describe our anaesthetic management of two patients with Pompe disease for a caesarean section.

## 1. Introduction

Pompe disease (PD), also referred to as acid maltase deficiency (AMD) or glycogen storage disease type II (GSDII), is an autosomal recessive disorder caused by a deficiency of the lysosomal enzyme acid-*α*-glucosidase (GAA) [1]. In PD, lysosomal glycogen accumulates in many tissues with skeletal, cardiac, and smooth muscle most prominently involved. Severity varies according to the age of onset; the degree and severity of skeletal, cardiac, and respiratory muscle involvement; and rate of disease progression. The combined incidence of all forms of PD is estimated to be 1 : 40.000 [2]. In general, disease severity is inversely related to residual acid-*α*-glucosidase activity.

Although PD is often classified into two separate phenotypes—infantile and late onset—based on age of onset of symptoms, PD is a clinical disease spectrum. Patients with infantile PD, present in the first few months of life with a hypertrophic cardiomyopathy, generalized muscle weakness and hypotonia. Without enzyme treatment death due to cardiorespiratory failure occurs within the first year of life. Late-onset PD can present at any age and is characterized by the absence of cardiac involvement and a less dismal short-term prognosis. Symptoms are related to progressive skeletal muscle dysfunction. Proximal lower limb and paraspinal trunk muscles are usually affected first, followed by involvement of the diaphragm and accessory muscles of respiration. As the muscle weakness worsens, patients often become wheelchair dependent and may require assisted ventilation. Respiratory failure is the usual cause of increasing morbidity and mortality.

Alglucosidase alfa (recombinant GAA (rhGAA), Myozyme/Lumizyme) has been shown to be effective in the treatment of patients with early- and late-onset PD [3]. Individual response to enzyme replacement therapy (ERT) is however variable and determined by many factors: age of presentation, the rate of disease progression, muscle fibre type, defective autophagy, underlying genotype, and the development of rhGAA specific antibodies.

In this case report we describe the anaesthetic management of two patients with PD for a caesarean section.

## 2. Case Description

### 2.1. Patient 1

A 41-year-old nulliparous female (1.73 m/67 kg) with PD had been treated with enzyme therapy for 18 months at time of presentation. Before starting enzyme therapy there was a marked deterioration in muscle function, which manifested as a limb girdle weakness and a decrease in respiratory function, for which she needed nocturnal mechanical ventilation. After starting enzyme replacement therapy, there was a significant improvement in muscle strength.

As her pregnancy progressed, she noticed a marked deterioration in her effort tolerance. She needed more sleep, her walking distance decreased, and she felt short of breath. At 20 weeks pregnancy, her lung function tests showed a functional vital capacity of 1.6 l (40% of normal predicted value) sitting and FVC of 1.1 l (26%) supine. These results were similar to her lung function results prior to her pregnancy and were therefore related to the increased metabolic and respiratory demands of pregnancy rather than deterioration in her muscle function.

Following a multidisciplinary discussion, it was decided to do a caesarean section at 38 weeks of gestation, under combined spinal-epidural anaesthesia (CSE). In the normal healthy population, there is an increased risk of pelvic floor problems (including faecal and urinary incontinence) with a vaginal delivery. The recently reported incidence of previously unrecognised and underreported gastrointestinal symptoms including malabsorption and diarrhoea in patients with PD was a reason to avoid a vaginal delivery [4, 5]. Furthermore, there may be glycogen storage in the uterus as well, with unknown consequences during vaginal delivery.

The patient received our routine antacid prophylaxis, for example, oral ranitidine, sodium citrate, and metoclopramide. Monitoring was done by pulse oximeter, ECG, and automatic noninvasive blood pressure, set to record every 2.5 minutes. Combined spinal-epidural anaesthesia (CSE) at the L3-4 interspace was performed with the patient being in the sitting position. 1.6 mL bupi 0.5%/sufentanil (6.4 mg hyperbaric bupivacaine and 1.6 mcg sufentanyl) was injected intrathecally. Voluven coload of 500 mL and a phenylephrine infusion at 0.1 mcg/kg/min were commenced. The patient was placed supine in left lateral tilt position. She put on her own CPAP mask to assist her own ventilation. Throughout the operation her oxygen saturation remained above 95%. An adequate sensory block was reached 13 minutes after spinal injection. The phenylephrine infusion was increased to 0.24 mcg/kg/min to maintain baseline mean BP (80 mmHg). A live male infant was delivered 10 minutes after incision. Oxytocin 5 IU was titrated slowly intravenously (iv) followed by an infusion of 2.5 IU/hour of syntocinon for 4 hours. Routine antibiotic prophylaxis of 1 gram cefazolin was also administered intravenously. Estimated blood loss was 300 mL. Postoperative analgesia included regular oral paracetamol (four times a day) and a continuous infusion of ropivacaine 0.2% + sufentanil 0.5 mcg·mL^−1^ 6.0 mL per hour via the epidural catheter. She was monitored on the high-care unit before returning to the obstetric ward. The patient was discharged with her healthy baby boy four days postpartum.

### 2.2. Patient 2

A 30-year-old nulliparous woman (1.69 m/68 kg) with PD was receiving enzyme therapy for almost 4 years. Besides her PD, her medical history included a nasal septum correction without any anaesthetic problems. Prior to her pregnancy her symptoms included breathlessness on exertion and a mild increase in limb girdle weakness. She was not dependent on nocturnal ventilation. Her lung function tests prior to her pregnancy showed diminished respiratory muscle function with a FVC: 2.86 L (74%) which decreased to a FVC: 2.11 L (54%) towards the end of her pregnancy. Furthermore, ancillary investigations showed a mild increase of hepatic enzymes, AST 122 U/L and ALAT 129 U/L. Many patients with PD show increased liver function values, which may reflect the presence of ongoing muscle damage, although the levels measured are not indicative of the severity of the disease [6]. For the same reasons as the previous patient, she was planned for an elective caesarean section under combined spinal-epidural anaesthesia at 38 weeks of gestation.

She received the same routine antacid prophylaxis. After securing intravenous access, noninvasive blood pressure, ECG, and oxygen saturation monitoring were established. CSE at the L3-4 interspace was performed in the sitting position and from a solution of 4 mL bupivacaine 0.5% glucose with 1 mL sufentanil 5 mcg·mL^−1^ 1.7 mL, representing 6.8 mg hyperbaric bupivacaine with 1.7 mcg sufentanil, was injected intrathecally. Crystalloid coload of 500 mL and a phenylephrine infusion at 0.23 mcg/kg/min were commenced. The patient was placed supine with a left lateral tilt. She needed 4 increments of 2.5 mL 1% ropivacaine over her epidural to reach a block at the T4 dermatome.

The phenylephrine infusion was increased to 0.37 mcg/kg/min to maintain a baseline mean BP (85 mmHg). A healthy male infant was delivered uneventfully. Routine antibiotic prophylaxis of 1 gram cefazolin (iv) was administered; oxytocin 5 IU (iv) was given as a slow bolus followed by an infusion of 10 IU over 4 h (iv). Estimated blood loss was 200 mL. Postoperative analgesia included a continuous epidural and oral paracetamol. She was monitored for some hours at the high-care unit before she returned to the obstetric ward. She was discharged in a good condition with her son three days after the caesarean section.

## 3. Discussion

PD was first linked in 1963 to an inherited deficiency of the lysosomal enzyme acid alpha-glucosidase (GAA). This enzyme is responsible for the breakdown of glycogen to glucose. GAA deficiency leads to the intralysosomal accumulation of glycogen, primarily in muscle cells, causing a progressive loss of muscle function [7].

Until 2006 there was, other than ventilatory support, no treatment for patients with PD. In 2006 enzyme replacement therapy with alglucosidase alfa (Myozyme) was approved for the treatment of babies with infantile-onset PD. This was the first time a specific treatment was available for PD. A recently published Late-Onset Treatment Study (LOTS) showed that alglucosidase alfa treatment, as compared with placebo, has a positive, though modest, effect on walking distance and pulmonary function in patients with late-onset PD and may stabilize proximal limb and respiratory muscle strength [8]. Due to the introduction of ERT, we may increasingly have to deal with patients with Pompe disease who become pregnant. To our knowledge there is only one case report on a patient with Pompe disease undergoing a caesarean section [9].

Little is known about the use of alglucosidase alfa during pregnancy. In animal studies, alglucosidase alfa is not transported across the placental barrier and is, therefore, probably not harmful to the fetus. Furthermore, continuation of ERT during pregnancy has become common practice in other lysosomal storage diseases such as Gaucher disease and Fabry disease [10, 11].

Pregnancy itself is risky for these patients. Due to the mass effect of the uterus and the increased minute ventilation during pregnancy, the respiratory muscle function can be further challenged. The first patient had, prior to her pregnancy, a greater reduction of her respiratory muscle function capacity than the second patient, necessitating the use of noninvasive ventilation during her caesarean section in the supine position.

In addition, in order to avoid the risk of urinary and faecal incontinence, patients with PD should be allowed to undergo an elective caesarean section. If possible, regional anaesthesia is the technique of choice and far superior to general anaesthesia for elective caesarean section both for mother and child [12]. We decided to give regional anaesthesia by means of CSE, so we had the opportunity to minimize the amount of local anaesthesia to the spinal compartment. A lower dose of intrathecal local anaesthetic is likely to reduce the incidence of spinal-induced hypotension and possibly the severity of its maternal effects, at the expense of a slower onset and a shorter duration of anaesthesia and increased risk of intraoperative pain [13]. So, the epidural catheter functioned both as a backup (during the caesarean section) and postoperative analgesia. The backup function was needed in the second patient, preventing the risk of intraoperative pain and the need for a conversion to general anaesthesia.
